# Functional roles and networks of non-coding RNAs in the pathogenesis of neurodegenerative diseases

**DOI:** 10.1186/s12929-020-00636-z

**Published:** 2020-04-07

**Authors:** Yi-Ying Wu, Hung-Chih Kuo

**Affiliations:** 1 Institute of Cellular and Organismic Biology, Academia Sinica, No. 128, Sec. 2, Academia Road, Nankang, Taipei, 11529 Taiwan; 2 Graduate Institute of Medical Genomics and Proteomics, College of Medicine, National Taiwan University, Taipei, Taiwan

**Keywords:** Noncoding RNAs (ncRNAs), MicroRNAs (miRNAs), Long non-coding RNAs (lncRNAs), Neurodegenerative diseases, Motor neuron diseases, Central nervous system (CNS), Huntington’s disease (HD), Parkinson’s disease (PD), Alzheimer’s disease (AD)

## Abstract

Recent transcriptome analyses have revealed that noncoding RNAs (ncRNAs) are broadly expressed in mammalian cells and abundant in the CNS, with tissue and cell type-specific expression patterns. Moreover, ncRNAs have been found to intricately and dynamically regulate various signaling pathways in neurodegeneration. As such, some antisense transcripts and microRNAs are known to directly affect neurodegeneration in disease contexts. The functions of ncRNAs in pathogenesis are unique for each disorder, as are the pertinent networks of ncRNA/miRNA/mRNA that mediate these functions. Thus, further understanding of ncRNA biogenesis and effects might aid the discovery of diagnostic biomarkers or development of effective therapeutics for neurodegenerative disorders. Here, we review the ncRNAs that have so far been identified in major neurodegenerative disease etiology and the mechanisms that link ncRNAs with disease-specific phenotypes, such as HTT aggregation in HD, α-synuclein in PD, and Aβ plaques and hyperphosphorylated Tau in AD. We also summarize the known lncRNA/miRNA/mRNA networks that participate in neurodegenerative diseases, and we discuss ncRNA-related treatments shown to delay disease onset and prolong lifespan in rodent models.

## Introduction

The mammalian genome contains sequences for both protein-coding RNAs (mRNAs) and non-coding RNAs (ncRNAs). Since proteins represent the primary functional output of genetic information, mRNAs have been far more well-studied than their non-coding counterparts. However, over the past decades, functional interrogation of ncRNAs has slowly expanded, deepening our understanding of many biological processes. The diverse catalog of known ncRNAs includes long non-coding RNAs (lncRNAs; longer than 200 nucleotides), circular RNAs (circRNAs; generated from pre-mRNA back-splicing), small noncoding microRNAs (miRNAs; around 21–25 nucleotides), and natural antisense transcripts (NATs; generated by transcription in the opposite direction to protein coding transcripts). Although most ncRNAs cannot be translated into protein, many function in the regulation of important biological processes by modulating transcription and post-translational modifications [reviewed in [1]]. Among the subtypes of ncRNAs, microRNAs are known to influence many aspects of metazoan biology primarily by mediating mRNA stability and preventing translation [reviewed in [2]]. NATs also interfere with stability of the corresponding sense transcript and affect protein translation and function. Notably, some NATs may be translated and function as dominant negatives. LncRNAs are reported to coordinate chromosome architecture and regulate gene transcription during development and human diseases [3, 4]. LncRNAs also post-transcriptionally regulate gene expression as miRNA decoys or by trapping mRNAs in nuclear bodies and stress granules. A subset of lncRNAs interferes with translation by disrupting ribosome recruitment and preventing protein phosphorylation [1]. Finally, circRNAs are generated from coding genes via back-splicing, i.e., joining of the downstream 5′SS with the upstream 3′SS of an exon. CircRNAs are relatively stable compared to their linear cognates due to a lack of 5′ caps and 3′ tails. Functionally, circRNAs may act as RNA or protein decoys to regulate RNA stability and protein function. The most well-known function of circRNAs is that of a miRNA sponge, which is defined as a scaffold containing multiple binding sites for a specific miRNA. Binding of an miRNA to the scaffold prevents interaction of the sequestered miRNA with its target RNA transcripts. Thus, many circRNAs can affect mRNA stability by regulating miRNA targeting. CircRNAs may also interfere with protein function, giving rise to unique peptides [5].

Neurodegenerative disease is broadly defined as the progressive atrophy of neurons and neural tissues. Loss of functional neurons may lead to impairments in cognition and/or movement. Since each neurodegenerative disease preferentially affects a defined population of neurons, unique clinical profiles are associated with the different conditions [6]. Although shared mechanisms may contribute to neuronal loss in multiple diseases, the distinct pathological profiles are caused by specific genetic mutations and/or toxic aggregation of particular proteins. Some well-known hallmarks of neurodegenerative diseases include mutant hungtingtin (mHTT) aggregates in Huntington’s disease (HD) [7], α-synuclein-associated Lewy bodies in Parkinson’s disease (PD) [8], Amyloid-β aggregation and hyperphosphorylated Tau in Alzheimer’s disease (AD) [9], and TDP43 proteinopathies in frontotemporal lobe dementia (FTLD) [10] and amyotrophic lateral sclerosis (ALS) [11]. Thus, clearance of neurotoxic aggregates is a major focus of phenotypic assays for drug development, including those that measure autophagy-lysosomal network function and ubiquitin-proteasome-mediated protein degradation. Other assays measure endpoints related to cellular viability or neuronal death, such as caspase-dependent apoptosis [12], oxidative stress-induced mitochondrial dysfunction [13], and neurotrophic factor expression [14]. Current platforms for modeling neurodegeneration include a combination of patient-derived induced pluripotent stem cells (iPSCs), cellular models and animal models, which all recapitulate at least some disease-relevant phenotypes [6]. A number of promising therapeutic leads have been identified using these platforms, including antibodies, small molecules and other chemically modified drugs [11, 15]. However, a lack of approved effective treatments is still the primary clinical challenge for managing neurodegenerative diseases.

**Fig. 1:**
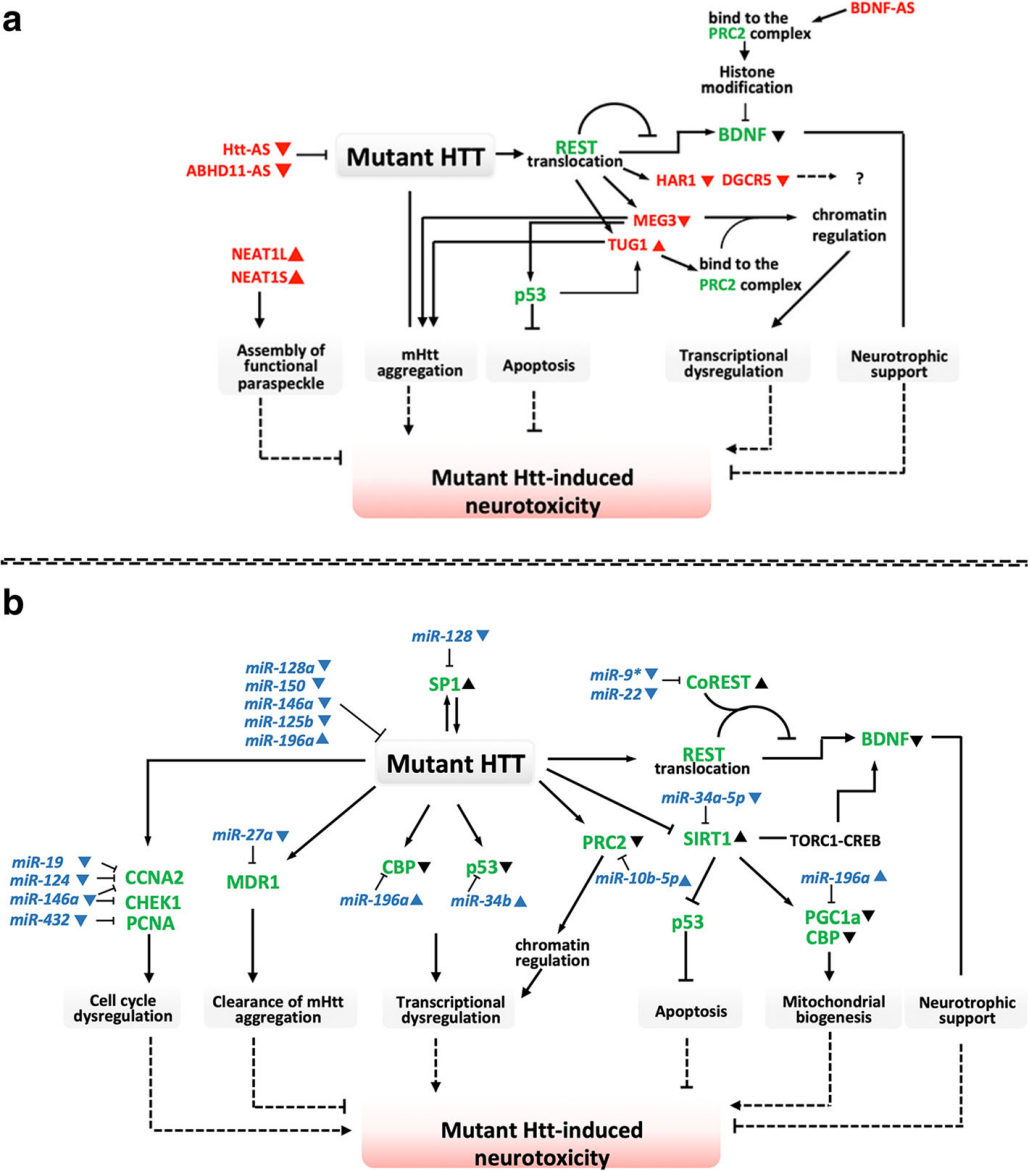
Cellular functions of ncRNAs involved in HD pathogenesis. (**a**) LncRNAs regulate mutant Htt induced neurotoxicity. (**b**) Networks of mutant Htt, microRNAs and mRNAs regulate neurotoxicity in HD. LncRNAs are shown in red, mRNA are shown in green and microRNAs are shown in blue

**Fig. 2:**
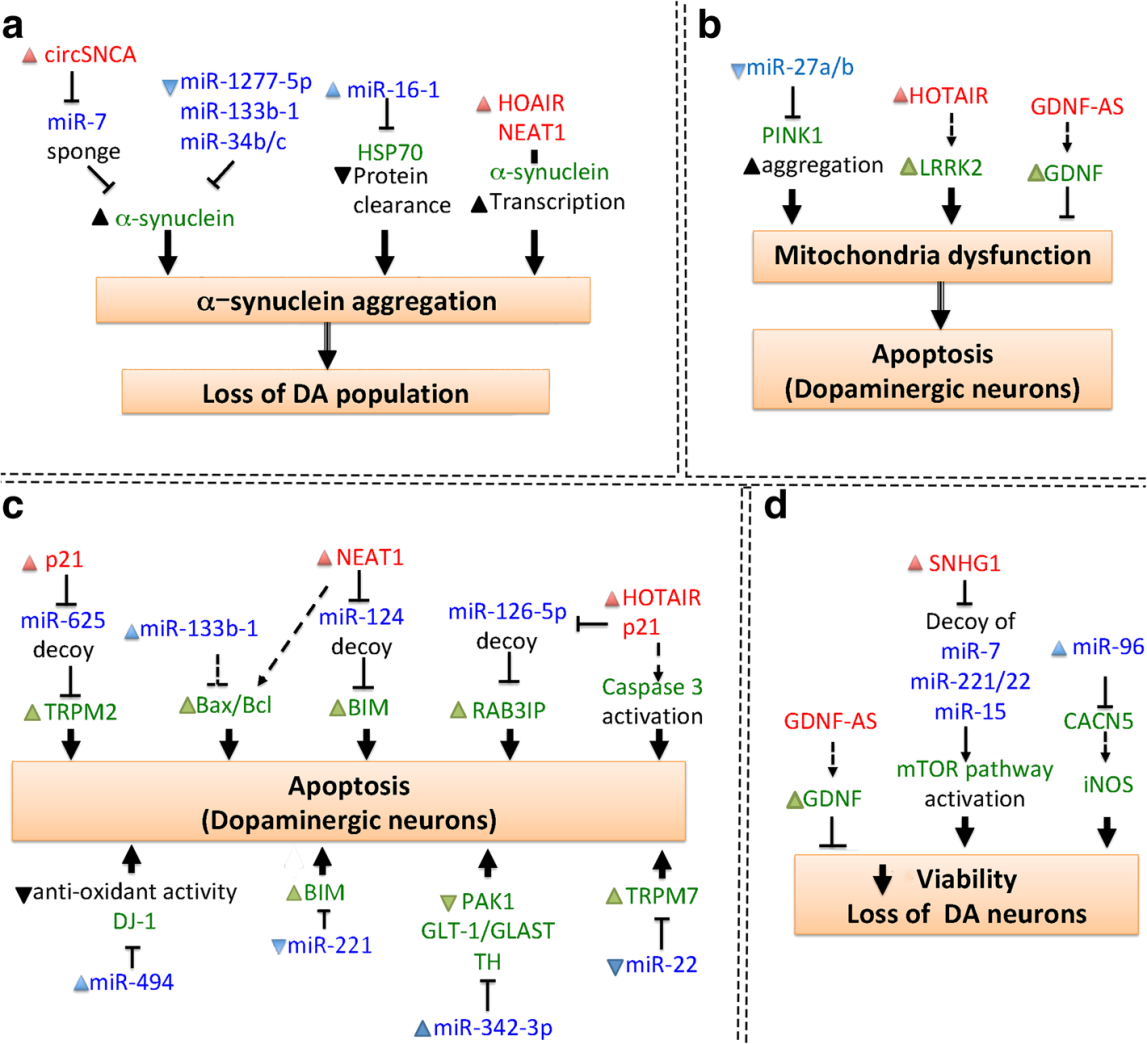
Cellular functions of ncRNAs involved in PD pathogenesis. (**a**) ncRNAs affect α-synuclein formation, which is linked to loss of dopaminergic (DA) neurons. (**b**) ncRNAs regulate apoptotic pathways leading to DA neuron degeneration. (**c**) ncRNAs regulate mitochondrial dysfunction. (**d**) ncRNAs regulates DA neuron viability

**Fig. 3:**
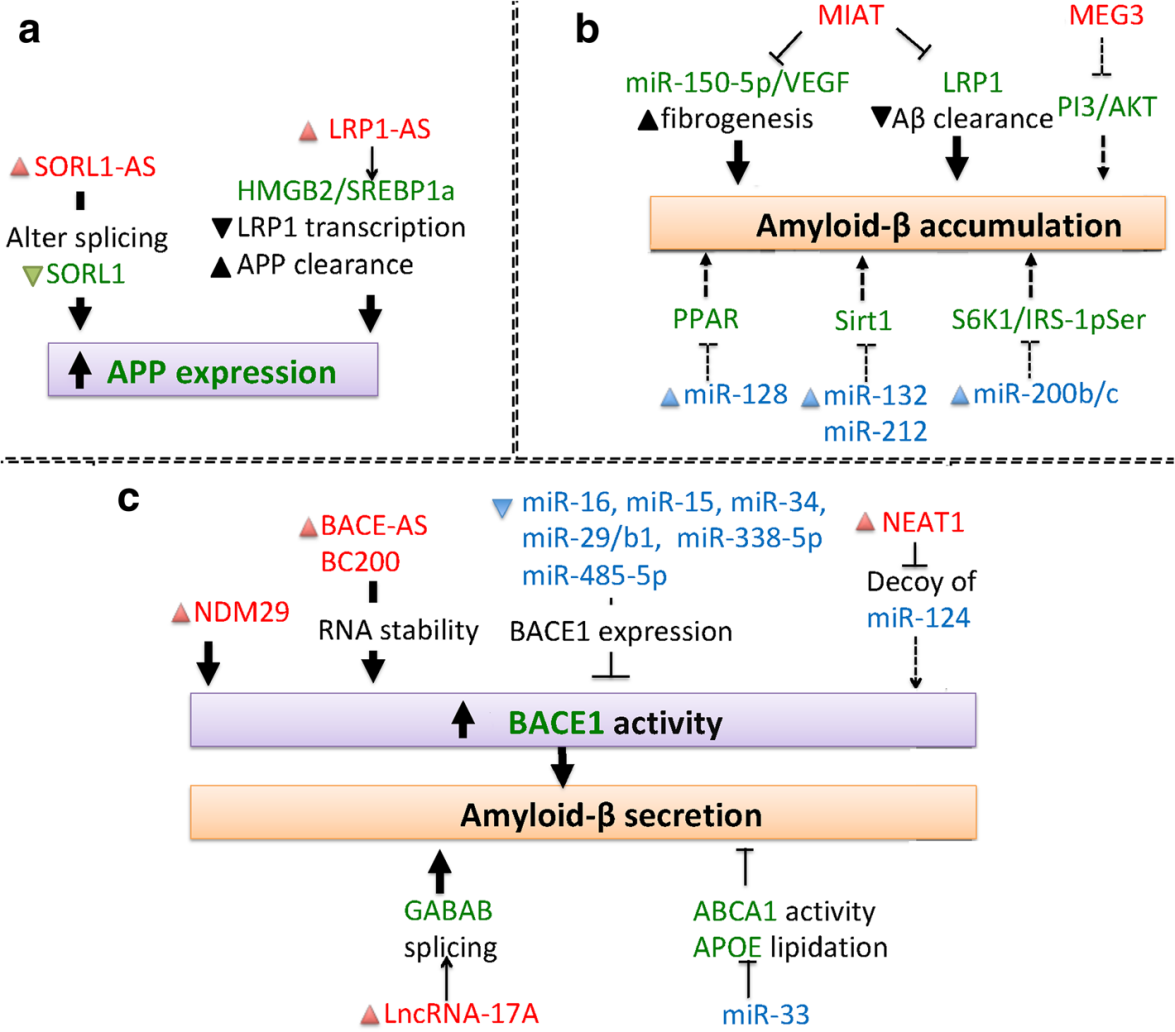
ncRNAs involved in APP expression and Aβ accumulation in AD. (**a**) ncRNAs promote APP expression (**b**) ncRNAs promote Aβ accumulation. (**c**) ncRNAs regulate BACE-1 function and Aβ secretion

**Fig. 4:**
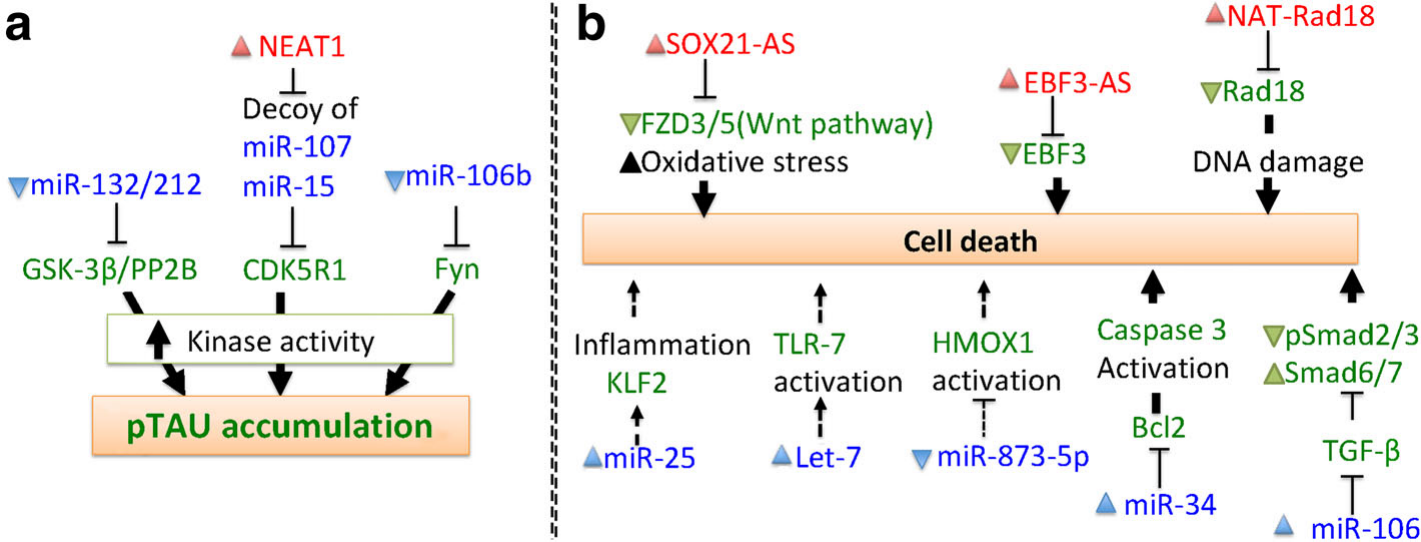
Cellular functions of ncRNAs that regulate pTau and cell death in AD pathogenesis. (**a**) ncRNAs promote kinase activity and pTau accumulation. (**b**) ncRNAs promote cellular pathways leading to neurodegeneration in AD

There is a growing list of ncRNAs that functionally associate with neural differentiation and function [16, 17], and it is thought that many ncRNAs and their related RNA networks may affect neurodegeneration [18]. As pathological mutations are linked to over-abundance/aggregation of certain proteins, ncRNAs have been studied in relation to distinct proteostatic events and pathogenesis in diseases, such as HD [7], PD [12] and FTLD and ALS [19]. ncRNAs have also been found to regulate shared signaling pathways, such as apoptosis, mitochondrial dysfunction and neurotrophic factor depletion in neurons and microglia [20–22]. Despite many exciting advances in ncRNA biology, the relationship between ncRNA and disease pathogenesis remains elusive. In this review, we survey the emerging roles of ncRNAs in adult-onset-associated neurodegenerative diseases, including HD, PD, AD, FTLD and ALS. We also summarize the current knowledge of how ncRNAs may affect disease-relevant mechanisms, such as HTT aggregation in HD (Fig. 1), α-synuclein aggregation in PD (Fig. 2), amyloid-β secretion and pTau accumulation in AD (Figs. 3 and 4).

### ncRNAs in HD pathogenesis

HD is a monogenic progressive neurodegenerative disease caused by expansion of a CAG repeat in the hungtingtin (*HTT*) gene. The expanded CAG repeat encodes a toxic polyglutamine tract that preferentially kills GABAergic projection neurons. Striatal medium spiny neurons (MSNs) are the major susceptible cell type in HD, and an extensive body of literature has shown recapitulation of HD phenotypes in mouse and cellular models after induction of HTT aggregation, REST-dependent BDNF depletion or mitochondrial dysfunction [6]. Many RNA species have been implicated in the etiology of HD as well (Table 1). Among these implicated RNAs, miRNA-mRNA networks are the most well-studied and clearly regulate phenotypes in HD models (Fig. 1a). In addition, several lncRNAs have been shown to regulate HD-related gene transcription via chromatin recruitment of repressors (Fig. 1b).

**Table 1:** ncRNAs and related processes in Huntington’s disease

	Name	up/down regulation	Description	model	Ref
lncRNA	*HAR1F, HAR1R*	down	*HAR1F* and *HAR1R* are direct targets of REST. Leading to thr repression of several important neuronal genes.	human HD brain_striatum	[23]
	*DGCR5*	down	*DGCR5* is a REST target lncRNA, which plays an important transcriptional regulatory role in HD	human HD brain	[24]
	*MEG3*	down	*MEG3* is a direct target of REST and found in the chromatin compartment of the cell in association with PRC2 complex	human HD brain	[25, 26]
	*MEG3, NEAT1*	up	Loss of function of *Meg3* and *Neat1* modulated mHtt aggregates and downregulation of the Tp53 expression	R6/2 mouse cortex	[27]
	*NEAT1-L, NEAT1-S*	up	*NEAT1* provides neuroprotection against mHtt-induced cytotoxicity (*NEAT1-L*) and oxidative stress-induced injury (*NEAT1-S*).	R6/2 mouse brain	[27-29]
	*TUG1*	up	*TUG1* is a direct downstream target of p53 and could modulate mHTT-induced cytotoxicity by p53 activation. *TUG1* could be able to binding to the PRC2 epigenetic regulatory complex.	human HD brain	[30]
	*TUNA*	down	*TUNA* expression was associated with pathological disease severity, decreasing significantly as the disease grade increased.	human HD brain_caudate nucleus	[31]
	*LINC00341*	up	unknown	human HD brain	[25]
	*RPS20P22*	up	*RPS20P22* regulates RPS20, which decreases will lead to accumulation of p53.	human HD brain	[25]
	*LINC00342*	down	unknown	human HD brain	[25]
NATs	*Abhd11-os*	down	*Abhd11-os* (called *Abhd11-AS1* in human) attenuate the mHtt-induced tocixity and contribute to striatal vulnerability in HD.	R6/2 mouse model	[32]
	*HTTAS_v1*	down	*HttAS-v1 *reduces endogenous mHtt tanscript level through RISC-dependent miR-like mechanism	human HD brain_frontal cortex	[7]
	*BDNF-AS*		*BDNF-AS* was associated with the recruitment of polycomb repressive complex 2 (PRC2), which locally induces the trimethylation of histone H3K27 within the locus and in that way plays an important role in the development of HD.		[25, 33, 34]
others	sCAGs	up	*sCAG* significantly decreased HTT-mediated neuronal viability in an Ago2-dependent mechanism.	R6/2 mouse striatum; human HD brain	[35]
miRNAs	miR-9*	down	miR-9* targets the components of the REST repressor complex, CoREST and modulating the neurotrophic genes expression.	HD patient	[26]
	miR-10b-5p	up	miR-10b-5p targets BDNF and correlated to aberrant polycomb repressive complex2 (PRC2) regulation.	HD brain_profrontal cortices	[36]
	miR-22	down	miR-22 was found to target HDAC4, Rcor1 (CoREST) and Rgs2 mRNA and achieve neuroprotection and inhibit neurodegeneration.	HD brain	[26]
	miR-27a	down	miR-27a increases MDR-1 expression; miR-27a can decrease the mHtt aggregates possibly through MDR-1 modulation.	R6/2 mice	[37]
	miR-34a-5p	down	miR-34a downregulated SIRT1 through binding the 3′ UTR of sirt1 mRNA.	R6/2 mice	[38]
	miR-34b	up	miR-34b is a P53-regulated miRNA and the levels may influence mHTT cytoplasmic distribution and toxicity	HD patient, plasma	[39]
	miR-214, miR-125b, miR-146a, miR-150	down	miR-214, miR-150, miR-146a and miR-125b target both huamn HTT and mouse Htt.	STHdh(Q111)/Hdh(Q111) cells	[40]
	miR-125b, miR-146a, miR-150	down	miR-146a, miR-150 and miR-125b are decreased in striatum of R6/2 mice. miR-125b and miR-150 target p53, which in turn regulates RelA/NFkB and miR-146a expressions	STHdhQ111/HdhQ111 cells	[41]
	miR-124	down	Decreased miR-124 expression could increase CCNA2 and is involved in deregulation of cell cycle	STHdhQ111/HdhQ111 cells	[42]
	miR-124a	down	The neuronal-specific miR-124 is dysregulated in HD tissues, probably as a result of increased repression by REST.	R6/2 mouse brain	[43]
	miR-128a	down	miR-128a targets the 3′ UTR of HTT, Huntingtin Interaction Protein 1 (HIP1) and SP1.	Transgenic HD monkeys	[44]
	miR132	down	The level of miR-132 associated with Ago2. miR-132 supplementation produced symptomatic improvement of motor function and lifespan.	R6/2 mice	[45]
	miR-212/miR-132	down	miR-212/miR-132 clusters are significantly associated with CAG length and implicated in neuronal survival and shows strongest down-regulation in the striatum.	HD transgenic mice	[35]
	miR-196a	up	miR-196a might improve mitochondrial function by the upregulation of CBP and PGC-1α to promote oxidation phosphorylation and reduce oxidative stress, which was consistent with the amelioration of cytotoxicity by miR-196a.	HD brain, striatum	[46]
	miR-196a	up	The higher expression of RAN binding protein 10 (RANBP10) in the brains of HD transgenic mice and exacerbates neuronal morphology and intracellular transport. miR-196a suppress the expression of RANBP10 through binding to its 3’UTR.	HD transgenic mice brain	[47]
	miR-196a	up	miR-196a decreased mHTT expression and pathological aggregates through alteration of several neuronal regulatory pathways including ubiquitin-proteasome systems, gliosis and cAMP response element-binding protein pathway.	HD-iPSC derived neurons	[48]
	miR-19, miR-146a, miR-432	down	The increase in the expressions of PCNA, CHEK1 and CCNA2 was found to be the result of decreased expressions of miR-432, miR-146a, and (miR-19a and miR-146a) respectively. Exogenous expressions of these miR-432 and miR-146a in HD cells rescued the abnormalities in cell cycle and apoptosis.	STHdh(Q111)/Hdh(Q111) cells	[43]

#### ncRNAs in the regulation of HTT expression/aggregation

mHTT aggregation is the primary phenotype of HD, and ncRNAs are known to regulate HTT expression and aggregation. Several ncRNAs directly regulate HTT transcripts to affect aggregation. For example, *HTTAS-v1*, the antisense RNA derived from *HTT*, regulates mHTT expression via dicer-dependent pathway and reduces the endogenous mHTT transcript level through a RISC-dependent microRNA-like mechanism. Because downregulated *HTTAS-v1* can be observed in HD brain sections, this NAT is thought to play a protective role in HD [7]. Certain microRNAs, including miR-214, miR-150, miR-146a and miR-125b, directly target the 3′UTR of both *HTT* and *mHTT*, which reduces HTT expression in human and mouse models [40, 49]. These microRNAs protect neurons, and miR-146a, miR-150 and miR-125b are downregulated in the striatum of the commonly used HD model, R6/2 mice [41]. Since miR-125b and miR-150 target p53, which regulates expression of the p65 subunit of NFκB (RelA/NFκB) and miR-146a, the regulatory mechanisms of miRNA-146a-, 125b- and 150-dependent HTT expression have been further linked to apoptosis in HD [41]. In transgenic HD monkeys, HTT is directly regulated by miR-128a, which targets the 3′UTR of *HTT*. Additionally, miR-128a targets transcription factors, Huntingtin Interaction Protein 1 (HIP1) and SP1, which promote *HTT* transcription. Thus, downregulation of miR-128a contributes to HTT related phenotypes by both direct and indirect mechanisms [44]. HTT transcription is also regulated by ncRNAs in HD models. For example, *ABHD11-AS* (MGI Symbol: Abhd11os), the antisense of *Abhd11*, reduces mHTT-mediated toxicity by altering mHTT transcription and was shown to contribute to striatal vulnerability in HD mouse models [32]. Moreover, ncRNAs are involved in defective HTT protein degradation in HD models. Clearance of mHTT by Multidrug resistance protein 1 (MDR1) is relevant to HTT aggression, and miR-27a is involved in MDR1 expression. The downregulation of MDR1, possibly by miR-27a, in HD increases HTT expression [37]. mHTT expression is also decreased by miR-196a through alterations in the ubiquitin-proteasome system, gliosis, cAMP response element-binding protein and several neuronal regulatory pathways in vivo. Since miR-196a confers protective effects in HD, its potential as a therapeutic target has been suggested [48].

#### ncRNAs in the regulation of REST activity and BDNF expression

Expression of Brain-derived neurotropic factor (BDNF) is downregulated in HD patients, and BDNF supplementation reduces mHTT-induced neurotoxicity in HD models. The BDNF antisense RNA (*BDNF-AS*) reduces BDNF expression via transcriptional repression, which involves polycomb repressive complex 2 (PRC2) recruitment to the BDNF locus and induction of histone H3K27 trimethylation. Thus, *BDNF-AS* is thought to play an important role in the development of HD [25, 33, 34]. In the prefrontal cortex of HD models, miR-10b-5p reduces BDNF expression through a direct interaction. Upregulation of miR-10b-5p is correlated to BDNF transcription with aberrant PRC2 regulation on the BDNF locus occurring by an unknown mechanism [36, 50]. Sirtuin 1 (SIRT1) is downregulated by miR-34a binding to the 3′ UTR of SIRT1 mRNA. Thus, the downregulation of miR-34a may provide a mechanistic link between BDNF expression and mitochondrial biogenesis [38].

In HD, BDNF depletion is linked to Repressor element 1-silencing transcription factor (REST)-dependent transcription regulation. REST is neuron-specific repressor of transcription, which is involved in demethylation of histone H3 lysine 4 (H3K4). The recruitment of a repression complex is an essential step in REST-mediated repression, and cofactors include SANT-domain-containing CoREST and BHC110 (also known as LSD1) demethylase, among others [51]. Normally, HTT prevents REST-mediated transcriptional repression by acting in a cytoplasmic complex with Huntingtin-associated protein 1 (HAP1). This function is lost in mHTT, allowing REST to assemble its repressor complex in the nucleus [52]. Thus, mHTT permits REST translocation from cytoplasm to nucleus, which then leads to BDNF transcription repression and depletion [53]. Human accelerated region 1 (HAR1) is a direct target of REST, and a reduction in HAR1F/R transcript levels leads to repression of several important neuronal genes with unknown functions in human HD striatum [23]. DiGeorge syndrome critical region gene 5 (*DGCR5*) is a lncRNA that is regulated by REST, and the downregulation of *DGCR5* in HD brain suggests it may play an important transcriptional regulatory role in HD development [24]. Maternally expressed 3 (*MEG3*) is also regulated by REST, and it associates with the PRC2 complex in chromatin. Downregulation of *MEG3* was reported in human HD brain and may modulate mHTT aggregation [25]. miR-9 and miR-22 were found to target several gene transcripts, including HDAC4, REST corepressor 1 (coREST) and Rgs2; these miRNAs were therefore linked to HD by repression of coREST expression, which normally promotes REST-mediated BDNF depletion [26]. As such, miR-22 is thought to be neuroprotective and inhibits neuronal loss in HD brain [54, 55]. The neuron-specific miR-124 is downregulated in HD mouse models, and REST was suggested to be a primary mediator of this effect [42]. Because miR-124 is associated with the cell cycle, REST appears to be involved in multiple mechanisms of HD pathogenesis [42].

#### ncRNAs in the regulation of HD-associated apoptosis

An expanded CAG repeat in HTT exon 1 is the cause of HD, and decreasing the length of expanded repeats has been shown to improve neuronal viability. Exogenous expression of 21-nucleotide small-CAG repeated RNA (*sCAG*) regulates cell viability, similar to small RNA extracted from HD brain. This sCAG treatment activates an Ago2-dependent mechanism by targeting CAG regions of the mHTT transcript, significantly decreasing mHTT-mediated neuronal death [35]. Cytotoxicity of mHTT can also be modulated by Taurine Upregulated Gene 1 (*TUG1*) via p53. Since *TUG1* also binds to the PRC2 epigenetic regulatory complex of genes, *TUG1* upregulation may be involved in multiple molecular pathways in the human HD brain [30]. Furthermore, both *MEG3* and *NEAT1* are upregulated in HD mouse R6/2 cortex, and reducing *MEG3* and *NEAT1* expression modulates mHTT aggregates and TP53 expression through unknown mechanisms [27–29]. *RPS20P22* regulates RPS20 expression, and the decrease of *RPS20P22* leads to accumulation of p53 in HD brain [25]. In addition, apoptosis of GABAegic neurons is regulated by several microRNAs in HD. For example, miR-34b regulates P53 expression, which may influence mHTT-mediated apoptosis. Upregulation of miR-34b in HD patient brain and plasma samples suggests it may also play a role in HD [39]. mHTT was shown to reduce miR-432, miR-146a, miR-19a and miR-146a in a cellular system. These miRNAs directly target cell cycle genes, such as PCNA, CHEK1 and CCNA2. Notably, cell cycle dysregulation is linked to the decreased expression of CCNA2, and exogenous expression of miR-432 and miR-146a in HD cells rescues cell cycle abnormalities and apoptosis [43].

#### ncRNAs in other mechanisms of HD pathogenesis

In the striatum of HD brains, reduced mitochondrial function is evidenced by low oxidative phosphorylation and high oxidative stress. Interestingly, miR-196a upregulation modulates mitochondrial function by its effects on CBP and PGC-1α, and targeting miR-196a was reported to restore mitochondrial function in HD [46]. Furthermore, miR-196a also targets the 3′UTR of RAN binding protein 10 (*RANBP10*), an action which enhances neuronal morphogenesis and intracellular transport in an HD mouse model [47]. In another example, the level of miR-132 is associated with Ago2-dependent HTT clearance in animal models of HD, and supplementation with miR-132 improves motor function and lifespan in an HD mouse [45, 56]. Although the mechanism is still unknown, *TUNA* expression is negatively associated with pathological disease severity, i.e., decreased *TUNA* is associated with increased disease grade in HD patients [31]. Moreover, in HD brains, the expression levels of *LINC00341* and *LINC00342* are significantly different in comparison with control subjects [25].

Taken together, mounting evidence shows that ncRNAs play many important roles in the development of HD, with much evidence coming from HD models (Table 1). Further understanding the functional roles of dysregulated ncRNAs in HD is expected help to unravel disease pathogenesis and identify potential therapeutic targets.

### ncRNAs in PD pathogenesis

PD is an adult-onset neurodegenerative disorder that affects about 1 % of people over 60 years of age. In this disease, a motor deficit is caused by selective loss of A9-type dopaminergic neurons that project from the substantia nigra in the midbrain to the dorsal striatum. Intra-neuronal aggregation of α-synuclein in Lewy bodies is a pathological hallmark of PD [8]. Approximately 10% of PD cases are inherited as genetic mutations of SNCA (α-synuclein), PARK2 (Parkin), PINK1 (PTEN-induced kinase 1), PARK7 (protein deglycase DJ-1), LRRK2 (leucine-rich repeat kinase) or ATP13A2 (ATPase type 13A). Most PD-causing mutations are in genes that regulate mitochondrial function and oxidative stress, and similarly, sporadic PD may be caused by certain environmental factors that disrupt mitochondrial function, such as pesticides and heavy metals. Thus, PD is often modeled with MPTP/MPP+ (1-methyl-4-phenyl-1, 2, 3, 6-tetrahydropyridine) or 6-OHDA (6-hydroxydopamine), which induce mitochondrial dysfunction and oxidative stress in dopaminergic projection neurons [57]. ncRNAs have been reported to disrupt mitochondrial function via effects on gene expression and α-synuclein aggregation (Table 2 and Fig. 2).

**Table 2:** ncRNAs and related processes in Parkinson’s disease

	Name	up/down regulation	Stimulation	Description	Model	Ref
lncRNAs	*HOTAIR*	up	MPTP	correlated with LRRK2 upregulation and activating caspase 3 dependent apoptosis	mouse model	[58]
	*HOTAIR*	up	MPTP	blocking miR-126-5p/RAB3IP interaction and promoting DA neurona death	mouse model	[59]
	*NEAT1*	up	MPTP	Positively correlated with treated MPTP concentration. NEAT1 upregulation promotes Bax/BCl ratio, caspase 3 activity and α-synuclein expression. *NEAT1* knokdown promotes cell viability and supresses apotosis.	mice, cell line	[60]
	*NEAT1*	up	MPTP	*NEAT1* correlated with neuroinflammation after MPP+ treatment (IL-1b, IL-6 and TNF-a upregulation). *NEAT1* serves as miR-124 decoy and promotes cell death and apoptosis.	mice, cell line	[61]
	*NEAT1*	up	MPTP	*NEAT1* promotes PINK1 protein stability via preventing degredation. *NEAT1* positively correlated to LC3-II/LC3-I level and promotes autophogy.	mice, cell line	[62]
	*NORAD*	down	MPP+	*NORAD* protects cell against MPP+ induced cytotixity inclduing caspase3/7, ROS and LDH activity with unknown mechanism.	cell line	[12]
	*p21*	up	MPTP	*p21* positvely regulates TRPM2 expression by targeting miR-625. p21 serves as a miR-625 decoy and inhibits TRPM2 function in causing neuronal injury.	cell line	[63]
	*p21*	up	MPTP	*p21* is miR-1277-5p decoy and prevents miR-1277-5p directly targeting a-synuclein expression. P21/miR-1277-5p/a-synuclein axis leads to apoptosis in MPP+ induced PD models.	mouse	[64]
	*SNHG1*	up	MPTP	*SNHG-1* is a miRNA sponge of miR-221/222 and prevents p27 targeting and activation of mTOR pathway. Downregulation of *SNHG1* attenuated MPP+ induced decreases in LC3-II (an autophagic marker) levels and cytotoxicity through the miR-221/222/p27/mTOR pathway.	cell line	[65]
	*SNHG1*	down		miR-15 decoy and inhibit miR-15 function	cell line	[66]
	*SNHG1*	up	LPS	Upregulation of *SNHG1* promotes neuroinflammation in BV2 microglia of PD models. *SNHG-1 *functioned as a competing endogenous RNA for miR-7 to regulate NLRP3 expression leading to the activation of NLRP3 inflammasome.	mice, cell line	[67]
	U1 splicesomeal lncRNA, RP11-462G22.1	up		Upregulated in patiensts’ leukocyte, amygdala and substantia-nigra. Computational prediction shows miRNA decoy, and exhibit a more complex secondary stem-loop structure. Potentially as decoy of 21 different miRNAs (potential ceRNA).	PD patient tissue	[68]
	tRNA-derived fragment			A list of tRNA-derived fragment are consistently founded in pateits’ CSF, serum and cortex. The tRNA-derived fragment are the biomarkers of PD.	PD CSF, cortex, serum	[69]
NATs	*UCHL1-AS*		MPTP	*UCHL1-AS *is an antisense to the mouse ubiquitin carboxy terminal hydrolase L1 and activates transcription of UCHL1. UCHL1 is under the regulation of Nurr1(a major transcription factor) in dopaminergic differentiation, maintenance and related to cellular stress in the brain.	mouse, PD patient	[25]
	*GDNF-AS*		6-OHDA	*GDNF-AS* is coded for GDNF and enable to promote GDNF expression by 2 fold. *GDNF-AS *delivery by Adeno-associated virus was able to ameliorate motor deficits and neurodegeneration of DA neurons in a PD mouse models lesioned by6-OHDA treatment.	mouse	[70]
circRNAs	*circSNCA*	up	MMP+	*circSNCA* serve as a miR-7 sponge and prevents miR-7 targeting SNCA expression. Increased SNCA induce pro-apoptotic genes (CASP3, BAX, PTEN and P53). Knockdown of circSNCA shows the anti-apoptotic gene (BCL2) expression and prevents apoptosis.	cell line	[12]
miRNAs	miR-126	up	6-OHDA	miR-126 directly targets p85b, IRS-1, SPRED1 and impaires IGF-1/PI3K/AKT signaling. miR-126 leads to 6-OHDA induced neurotoxity.	cell line	[71]
	miR-126-5p	down		miR-126-5p increase cell proliferation and reduce apotosis via targeting RAB3IP	cell line	[59]
	miR-133a/b			miR-133 targeted PITX3 mutations/polymorphisms are not related to PD risk.	human genome sequence	[72]
	miR-133b	down		miR-133b targets RhoA (inhibitor of axonal growth) and indcue axonal outgrowth. miR-133b inhibits a-synuclein expression, Bcl/bax ratio and activates pAkt for neuronal survival.	cell line	[72]
	miR-153	down	MPP+	miR-153 reduces p38 activation and prevents neuro-inflammation induced apoptosis	mouse primary cortical neuron	[73]
	miR-16-1	up		miR-16-1 targets Hsp70 3’UTR and negatively regulate a-synuclein aggregation	SH-SY5Y	[74]
	miR-183	up		miR-183 promotes apotosis of substantia nigra neuron by inhibit the expression of OSMR.	cell line	[75]
	miR-205	down		mir-205 suppresses the expression of LRRK2 protein through a conserved-binding site at the 3′-UTR of LRRK2 gene and promotes neurite outgrowth.	PD brain section	[76]
	miR-22	down	6-OHDA	miR-22 overexpression downregulates the level of TRPM7, exhibited neuroprotective and reversal effect on the 6-OHDA-induced PCL2 cell growth and apotosis.	cell line	[77]
	miR-221	down	MPP+	DJ-1 may increase miR-221 expression through the MAPK/ERK pathway, leading the repression of apototic molecules BIM.	cell line	[70]
	miR-27a/b	down	CCCP	miR-27a/b suppress PINK1 expression through targeting 3′ UTR. Donwregulated PINK1 by miR-27a/b prevents its aggregation upon mitochondria damage and inhibits lysosomal degradation of damaged mitochondria.	cell line	[21]
	miR-342-3p	up	MPTP	miR-342-3p directly targets PAK1(p21-activated kinase 1) in Wnt signallg pathway. miR-342-3p also reduce expression of GLT-1, GLAST, and leads to reduce TH expression and the related apoptosis.	mouse	[6]
	miR-34b/c	down		miR-34b/c targets α-synuclein 3’UTR and reduces expression. In SNP of α-synuclein in Parkinson’s disease, failed miR-34b/c targeting causes α-synuclein expression and the related PD pathogenesis.	cell line	[78]
	miR-494	up	MPTP	miR-494 directly binds to 3′-UTR of DJ-1 transcript and inversely regulates the expression of DJ-1 which compromise anti-oxidative defence of cell.	mice	[79]
	miR-7	down		miR-7 represses a-synuclein protein level through binding 3′ UTR of A4 component of amyloid precursor (SCNA; gene name of a-synuclein) and protects cell against oxidative stress.	PD brain section	[12]
	miR-7/miR-153	down	MPP+	Both miR-7 and miR-153 activate p70S6K/pS6RP/SAPK/JNK mediated mTOR pathway.	mouse primary cortical neuron	[73]
	miR-96	up	MPTP	CACNG5 is the target gene of miR-96. CACNG5 and BCL2 inhibition are linked to the activation of iNOS and apotosis.	mice	[80]

#### ncRNAs in the regulation of α-synuclein expression and Lewy body formation

α-Synuclein upregulation is a crucial event that promotes its aggregation in Lewy bodies, and several ncRNAs have been found to regulate α-synuclein expression and aggregation in PD (Fig. 2a). The lncRNA, *NEAT1*, promotes transcription of α-synuclein, and it also enhances the Bax/Bcl ratio and caspase 3 activity in PD; thus *NEAT1* appears to participate in α-synuclein-associated apoptosis [60]. Several downregulated microRNAs in PD patient tissues are linked to α-synuclein upregulation and disease phenotypes. The downregulation of miR-34b/c prevents its targeting of the 3′UTR of α-synuclein, leading to α-synuclein upregulation. Moreover, it has been proposed that single nucleotide polymorphisms (SNPs) in α-synuclein reduce the binding capacity of miR-34b/c to promote α-synuclein-related PD pathogenesis [78]. Heat shock protein (HSP) 70 participates in the clearance of α-synuclein aggregation, and miR-16-1-mediated HSP70 downregulation has been linked to increased α-synuclein aggregation [74]. Another microRNA, miR-133b, also decreases α-synuclein expression, the Bax/Bcl ratio and pAkt activation to promote neuronal survival [20]. It was further reported that miR-133b targets RhoA to disinhibit axonal outgrowth. Notably, whole genome sequencing of PD patients failed to identify mutations/polymorphisms in miR-133b that are related to PD risk [72]. Thus, treatment with exogenous agents to increase miR-133b represents a potential therapeutic strategy for PD.

ncRNA networks have also been implicated in PD pathogenesis. For example, miR-1277-5p directly targets α-synuclein (*SCNA*), reducing its expression. *LncRNA-p21* is a decoy for miR-1277-5p and prevents the targeting of SCNA mRNA. Interestingly, the p21/miR-1277-5p/α-synuclein RNA network is required for apoptosis in models of MPP + -induced PD [64]. Furthermore, it was reported that miR-7 represses α-synuclein protein expression by binding the 3′UTR of SCNA, an action that protects cell against oxidative stress [12]. The circRNA, *circSNCA*, is derived from SNCA mRNA CDS and serves as a miR-7 sponge. The upregulation of *circSNCA* therefore promotes α-synuclein expression and pre-apoptotic gene expression (CASP3, BAX, PTEN and P53) in PD models. Thus, the *circSNCA/miR-1277-5p/α-synuclein* network positively regulates α-synuclein expression. In line with this notion, downregulating *circSNCA* expression rescues cell death after pramipexole (PPX) treatment [12].

#### ncRNAs in the regulation of PD-associated mitochondrial dysfunction

Mitochondria dysfunction is a major cause of Parkinsonism and degeneration of dopaminergic neurons [81]. ncRNAs have also been found to regulate mitochondrial dysfunction, which leads to a loss of dopaminergic neurons (Fig. 2b). Dysregulation and aggregation of PTEN-induced kinase 1 (PINK1), a mitochondrial kinase, has been implicated in PD pathology. When animals are treated with MPTP, the molecule is selectively taken up by dopaminergic neurons, inducing their degeneration via impaired mitochondrial function. *NEAT1* upregulation is positively correlated with MPTP concentration, and promotes PINK1 protein stability. *NEAT1* also promotes autophagy, as evidenced by an increased LC3-II/LC3-I level in MPTP-treated mice [62]. Autophagy is an essential pathway of degrading cytoplasmic protein aggregates and defective organelles via targeting to lysosomes. Imbalances of autophagy are linked to the retention of dysfunctional mitochondria in cells, which contributes to neurodegeneration in PD. As PINK1 is essential for the induction of mitochondrial autophagy, mutations in PINK1 have been linked to autophagy imbalances and PD [21, 62]. Several microRNAs that target PINK1 have been identified in PD models. For example, PINK1 can be reduced by direct targeting with miR-27a/b, which inhibits lysosomal degradation of damaged mitochondria [21].

Protein deglycase DJ-1 (parkin7) is essential for combatting oxidative stress in PD. Functionally, DJ-1 protects neurons from apoptosis by increasing miR-221 expression, leading to repression of proapoptotic BIM. Although the direct target of miR-221 is unknown in PD, DJ-1 potentially regulates miR-221 transcription via the MAPK/ERK signaling pathway [70]. In another example, the DJ-1 transcript is targeted by miR-494 and inversely regulates DJ-1 expression, compromising the antioxidant defenses of the cell [79].

Dysregulation of leucine-rich repeat kinase 2 (LRRK-2) is one of the most common PD-linked effects on kinases. In the MPTP-treated mouse model, lncRNA *HOTAIR* (Hox transcript antisense intergenic RNA) is upregulated, along with LRRK2 upregulation and induction of caspase 3-dependent apoptosis. Reducing *HOTAIR* can rescue dopaminergic neuron degeneration and reduce LRRK2 expression by some unknown mechanism [58]. miR-205 targets the 3′UTR of LRRK2 and suppresses its expression. MiR-205 downregulation is observed in brains of PD patients, including those with either sporadic disease or those with familial PD derived from the LKKR2(R1441G) mutation. Overexpression of miR-205 can reduce LRRK2 expression and promote neurite outgrowth in cell models [76].

#### ncRNAs in the regulation of PD-associated apoptosis

As the loss of dopaminergic neurons is responsible for symptoms in PD patients, signaling pathways that control cytotoxicity may be targetable to prevent neuronal loss (Fig. 2c and d). LncRNA activated by DNA damage (*NORAD*) is an exonic transcript from Chr20q11.23 that sequesters PUMILIO proteins to promote genome stability. In MPP + -treated cells, *NORAD* is downregulated, promoting cytotoxicity, caspase3/7 activation, ROS production and LDH release. Lentivirus-expressed *NORAD* protects cell against MPP+ cytotoxicity by unknown mechanisms [12]. Another category of ncRNAs, tRNA halves (*tRHs*), are tRNA-derived fragments (*tRF*s) that have been implicated in cell stress and neurodegeneration in PD tissues [68]. Moreover, several microRNAs are known to regulate PD-associated apoptosis by directly repressing gene expression. miR-126 is involved in regulating neuronal toxicity and cell death processes. In 6-OHDA-treated PD cell models, miR-126 is upregulated and directly targets p85b, IRS-1, SPRED1 to suppress IGF-1/PI3K/AKT signaling. Along with other mechanisms, this action leads to neurotoxicity [71]. MiR-96 is upregulated in MPTP-treated PD mouse models, where it targets CACNG5. CACNG5 and Bcl2 inhibition are linked to the activation of iNOS and apoptosis [80]. In addition, upregulation of miR-342-3p can be detected in MPTP-treated mice. MiR-342-3p directly targets PAK1 (p21-activated kinase 1) in the Wnt signaling pathway and also reduces expression levels of Glutamate transporter subtype 1 (GLT-1) and L-glutamate/L-aspartate transporter (GLAST). These actions impair neuronal viability and lead to apoptosis [6]. In 6-OHDA-treated cell lines, miR-22 is downregulated. Exogenous overexpression of miR-22 markedly downregulates TRPM7, inhibiting neurotoxicity and rescuing cell growth [77]. Downregulation of miR-126-5p has also been implicated in causing PD-like toxicity in cell lines. As miR-126-5p increases cell proliferation and reduces apoptosis by targeting RAB3IP, this microRNA can protect neurons from apoptosis [59]. In MPP + -treated cells, both miR-7 and miR-153 are downregulated. The p70S6K/pS6RP/SAPK/JNK axis of the mTOR pathway is inactivated by both miR-7 and miR-153 by unknown mechanisms [73]. Moreover, miR-183 upregulation promotes apoptosis of neurons in the substantia nigra and inhibits OSMR expression [75].

Recently, studies on the more complex RNA networks (i.e., lncRNA/microRNA/mRNA) have revealed further effects on apoptosis in PD. In MPP + -treated mice, *HOTAIR* promotes apoptosis and reduces cell viability. Knockdown of *HOTAIR* protects from death of dopaminergic neurons by inhibiting caspase 3 activity. *HOTAIR* prevents microRNA-126-5p from targeting RAB3IP. As RAB3IP promotes autophagy and is involved in neuronal death in PD, this action promotes apoptosis. The *HOTAIR*/miR-126-5p/RAB3IP axis is linked to PD progression and its disruption has been suggested as a therapeutic target for PD [59]. Small nucleolar RNA host gene 1 (*SNHG1*) is gradually upregulated in MPP + -treated cells and animal models. Knockdown of *SNHG1* reduces LC3-II expression and MPP + -induced cell death. The microRNA-221/222 cluster is also associated with PD by virtue of its downregulation in blood samples of PD patients. MicroRNA-221/222 facilitates LC3-II formation and reduces MPP + -induced neurotoxicity. *SHNG1* can serve as a miRNA sponge for miR-221/222 and prevents targeting of p29, a key regulator of mTOR phosphorylation and cell death [65]. LncRNA-*p21* upregulation also promotes caspase 3 activation and increases Bcl family-initiated apoptosis. TRPM2, a nonselective Ca^2+^-permeable channel, is upregulated in the brain of PD patients [82]. It has been shown that miR-625 directly targets TRPM2 to prevent its expression. LncRNA-*p21* serves as a sponge for miR-625, and thus, it promotes TRPM2 expression. This *p21*/miR-625/TRPM2 regulatory network has also been linked to PD pathogenesis [63].

#### ncRNAs involved in other mechanisms of PD pathogenesis

Glial cell-derived neurotrophic factor (GDNF) deficiency has been demonstrated in PD models, and exogenous GDNF promotes dopaminergic neuron survival. *GDNF-AS* is coded for by GDNF mRNA and promotes GDNF expression by 2-fold. *GDNF-AS* delivery by Adeno-associated virus (AAV) ameliorated motor deficits and neurodegeneration of dopaminergic neurons in a mice with unilateral 6-OHDA lesions [70]. *UCHL1-AS* is an antisense RNA to ubiquitin carboxy terminal hydrolase L1 (UCHL1) that activates its translation. UCHL1 is under the regulation of Nurr1 (a major transcription factor) and functions in dopaminergic neuron differentiation and maintenance as well as cellular stress response in the brain. UCHL1 is mutated in a rare form of early-onset PD and loss of activity is responsible for disease. Overexpression of *UCHL1-AS* has been suggested as a method to promote translation of UCHL1 and prevent neurodegeneration [69].

LncRNA-*p21* promotes neuroinflammation by mechanisms including IL-1β, IL-6 and TNF-α upregulation. In MPP + -treated primary neurons, miR-153 is downregulated, which results in increased p38 activation and promotion of neuroinflammation-mediated apoptosis [73]. *NEAT1* is also correlated with neuroinflammation after MPP+ treatment, based on its upregulation of IL-1β, IL-6 and TNF-α. An RNA immunoprecipitation assay revealed that *NEAT1* serves as a miR-124 sponge and promotes apoptosis in MPTP-treated cells [61]. In BV microglial cells, *SHNG1* is upregulated during lipopolysaccharide-induced inflammation. Silencing *SHNG1* expression promotes miRNA-7 targeting of nod-like receptor protein 3 (NLRP3) expression and leads to activation of NLRP3 neuroinflammation. Thus, the action of *SHNG1* as a miRNA sponge for miRNA-7 promotes neuroinflammation in BV2 microglia [66].

In addition to the involvement of lncRNAs and microRNAs, tRNA-derived fragments (*tRF*s) are consistently founded in the CSF, serum and cortex of PD patients. Thus, the profiles of tRFs can be potentially used as biomarkers of PD [68]. Transcriptome analyses of leukocytes and brain samples from PD patients have been used to identify several PD-associated lncRNAs. For example, U1 splicesomal lncRNAs and RP11-462G22.1 are upregulated in patient leukocytes, amygdala and substantia nigra. Computational predictions show these lncRNAs may function as protective miRNA decoys and prevents the functioning of their complementary miRNAs. These two lncRNAs can potentially serve as decoys for 21 different miRNAs, suggesting they may act as competing endogenous RNAs (ceRNAs) to regulate RNA transcription during PD pathogenesis [67].

### ncRNAs in AD pathogenesis

AD is the most common cause of dementia in the elderly and affects the human population worldwide. Brain volume reductions in AD mainly result from hippocampal degeneration, and the major pathological features of this disease include extracellular amyloid-β (Aβ) plaques and hyperphosphorylated Tau in neurofibrillary tangles. AD patients often suffer progressive memory loss and acute cognition deficits during late-stage disease. Here we summarize the recent studies showing the involvement of ncRNAs in AD models; together, these studies suggest that ncRNAs play varied and important roles in the pathogenesis of AD (Table 3, Figs. 3 and 4).

**Table 3:** ncRNAs and related processes in Alzheimer’s disease

	Name	up/down regulation	genetic mutation	Description	Model	Ref
LncRNAs	*BC1*	up	Tg2576-APPswe	Induces APP mRNA translation via association with fragile X syndrome protein (FMRP). Induces Aβ peptides accumulation and affects spatial learning and memory impairments of mice.	mouse	[83]
	*BC200*	up		Regulate cell vialbility via directly targeting BACE1 mRNA expression. BC200 increases BACE1 expression and enhances Aβ1–42 expressioin.	AD brain section	[84–86]
	*LncRNA -17A*	up	Aβ1–42 treatment	Upregulates autophagy, deactivates GABAB signaling and induces neurodegeneration.	neuroblastoma	[87]
	*LncRNA -17A*	up	Inducing inflammation response	Impaires GABAB signaling through mediating receptor isoform switch via altering alternative splcing, and further induce Aβ secretion and increment of Aβ42/40 ratio.	AD brain section, SH-SY5Y cells	[88]
	*MEG3*	up	microinjection of Aβ25–35	Inhibiting the PI3K/Akt signaling pathway and apoptosis of hippocampal neurons, decreased Aβ expression, inhibited oxidative stress injury and inflammatory injury.	rat	[89]
	*MIAT*	down	APPswe/PSEN1ΔE9	Regulates amyloid clearance via regulating low-density lipoprotein receptor related protein 1 (LRP1) expression and miR-150-5p/VEGF mediated fibrillogenesis; Increased Aβ40 and Aβ42 levels and neuronal loss; Decreased brain microvessel number and the expression of tight junction proteins.	mouse	[90]
	*NDM29*	up	–	Induces APP synthesis and promotes cleavage activity of BACE1 and γ-secretase. Increase Aβ secretion and increment of Aβ42/40 ratio.	AD brain section, mouse neuroblastoma	[88]
	*NEAT1*	up	Aβ1–42 treatment	Mediates Aβ and pTau induced neuronal death via acting as miR-107 decoy.	human cell line	[91]
	*NEAT1*	up	–	Mediates Aβ secretion and pTau via regulating miR-124/BACE1 regulation.	mouse model	[92]
	*NEAT1*	up	–	Negetively regulating CDK5R1 mRNA level through positively regulating miR-15/107.	AD brain	[93]
	*P3Alu/SINE*	up in RPE	–	Neurodegeneration via P3Alu-induced inflammasomes (in RPE; still a hypothesis in neurons)	AD brain section, mouse model	[94]
NATs	*BACE1-AS*	up	APP-KM670/ 671NL and V717F	Stabilizes BACE1 mRNA and prevents miR-485-5p targeting on BACE1 mRNA. Increased Aβ42 is identified in the models.	AD brain section, mouse	[95, 96]
	*BDNF-AS*	up	–	In epigenetic level, promots BDNF depletion.	mouse model, HEK293T	[33]
	*EBF3-AS*	up	APPswe/PSEN1ΔE9, Aβ25–35 treated cell	Reduces EBF3 (early B cell factor 3) expression and promots cell death.	mouse model, cell	[97]
	*SORL1-AS(51A)*	up	–	*SORL1-AS* is the antisense orientation in intron 1 of the SORL1 gene and decrease SORL expression via altering splicing. SORL1-AS overexpression results in impaired processing of APP and increased Aβ formation.	AD brain section	[98]
	*SOX21-AS1*	up	Aβ1–40 treatment	Reducing Frizzled 3/5 (FZD3/5) mediated Wnt signaling pathwa and trigged oxidative stree and cell death in hippocampal neurons.	mouse model	[99]
	*NAT-RAd18*	up	Aβ1–40 treatment	Promotes DNA damage via reducing Rad18 expresion, and leads to cell death.	Rat cortical neurons	[100]
	*LRP1-AS*	up	–	Disrupts LRP1 mediated Aβ clarance via directly binding to high-mobility group box 2 (Hmgb2) and blocking the Srebp1a-dependent transcription of LRP1. Promotes APP endocytic trafficking, increases Aβ formation and decreases Aβ clearance.	AD brain section, Hmgb2 KO mice and mouse RAW264.7 cell line	[101, 102]
	*EBF3-AS, HAO2-AS, AD-lic1, AD-linc2*	up	–	Promoting neuron apoptosis.	AD brain section	[103]
microRNAs	let-7b	up	–	Activating RNA-sensing Toll-like receptor 7 and neuronal death.	AD CSF, mouse model, macrophages	[104]
	miR-106b	up	Tg-APPswe/PSΔE9	Regulate TGF-β signaling pathways and reduce phosphprylation of Smad2/3 and smad6/7 for promoting neurodegenration.	mouse models	[105]
	miR-106b	down	–	Regulates tau phosphoryation via targeting Fyn (tyrosine kinase) and increase pTau.	AD brain section, human cell line	[106]
	miR-128	up	3xTg-AD	Reducing APP expression, Aβ production and inflammatory response via tardeting peroxisome proliferator-activated receptor gamma (PPARγ) expression.	mouse model	[107]
	miR-34a, mIr132/212	down	3x Tg-AD (PSEN1(PS1M146V), APP (APPSwe) and Tau (P301L)	Correlated with Aβ production via targeting Sirt. Sirt can regulate Aβ production.	mouse model	[108, 109]
	miR-132/212	down	3x Tg-AD (PSEN1(PS1M146V), APP (APPSwe) and Tau (P301L)	Regulating Tau expression via direct interaction and association with GSK-3β and PP2B mediated tau phosphorylation.	AD brain section, mouse model, mouse neuroblastoma	[110]
	miR-142a-5p, miR-146a-5p, miR-155-5p, miR-455-5p	up	APPswe/PS1L166P, THY-Tau22	May be part of a protective response in AD.	AD brain section, mouse model	[110]
	miR-15/107	down	–	Increase expression of CDK5R1/p35 and consequently enhance CDK5 activity. miR-15/107 also modulates BACE1 expression and increase APP protein expresin and pTau formation.	AD brain section, human cell lines	[111, 112]
	miR-16	down		Attenuate Aβ mediated neurotoxicity via reducing BACE1 expression.	post morten tissue	[113]
	miR-200b/c	up	Aβ treatment/ Tg2576 transgenic mice	miR-200b/c inhibits S6K1-dependent phosphorylation of IRS-1, suppresse IRS-1pSer signaling pathway and cause insulin resistance in the brain. Aβ secretion is corelated in the models.	mouse model, mouse cell lines	[114]
	miR-25	up	Aβ1–42 induction	Downregulating KLF2 via Nrf2 signaling pathway to suppress proliferation and promot apoptosis.	mouse model	[115]
	miR-29a/b-1	down	–	Decreases BACE1 expression.	AD brain section	[116]
	miR-29c	down	–	Regulating the expression of BACE1 by directly targeting its 3’UTR and promoting cell proliferation via PKA signaling. Increasing BACE1 level via PKA/CREB signaling pathway.	Peripheral blood of AD pateints, SAMP mouse model	[117]
	miR-33	deletion	miR-33(−/−); APPswe/PSEN1ΔE9,	Increasing ATP-binding cassette transporter A1 (ABCA1) expression, ApoE lipidation, and decreaseing Aβ level.	mouse and human neural cells	[118]
	miR-34a	up	APPswe/PSEN1ΔE9	Regulating γ-secretase activity, BACE1 expression and lead to increasing Aβ level. Interacting with the 3′-UTR of bcl2 mRNA and inhibiting bcl2 translation, and increasing caspase 3 activity.	mouse model	[119, 120]
	miR-485-5p	down	–	miR-485-5p is corelated to BACE1 upregulation. The expression of two competitively regulatory RNAs, miR-485-5p and BACE1-AS, are dysregulated.	AD brain section	[96]
	miR-873-5p	down	Aβ1–42 induction	Preveting apoptosis via targeting Heme oxygenase 1 (HMOX1) expression level.	mouse model, rat cell line	[121]
	miR-338-5p	down	5XFAD transgenic (TG) mice	Mediaing amyloid formation via targeting BACE1; associtaed with NF-kB signaling pathway activation. Doenregulated miR-338-5p increases BACE1 expression, Aβ formation, and neuroinflammation.	AD brain section, mouse	[122]

#### ncRNAs in the regulation of Aβ generation and accumulation

It is well documented that production of the Aβ peptide is followed by secretion/release and extracellular precipitation. The Amyloid precursor protein (APP), a transmembrane protein, is processed by sequential cleavage by β-site APP cleaving enzyme-1(BACE1) and γ-secretase, to produce Aβ [123]. Several ncRNA species regulate extracellular accumulation of Aβ in AD models by either promoting translation (Fig. 3a), disrupting clearance (Fig. 3b) or modulating secretase cleavage (Fig. 3c).

The ncRNA, *BC1*, induces APP mRNA translation by associating with a fragile X syndrome protein (FMRP), and downregulation of *BC1* was found to induce Aβ peptide accumulation and spatial learning and memory impairments in transgenic mice carrying mutant APP [83]. LncRNA-*17A* is mapped to the intron of G-protein-coupled-receptor 51 (GPR51). The upregulation of LncRNA-*17A* is linked to Aβ secretion and elevation of Aβ42 production. Aβ42 is more toxic to neurons than Aβ40, and the secreted Aβ42 /40 ratio is associated with high risk of AD in patients samples [124]. Moreover, treatment with Aβ42 can stimulate lncRNA-*17A* expression to promote autophagy, neurodegeneration and deactivation of GABAB signaling, which occurs due to a GABAB receptor isoform switch based on alternative splicing [87, 88]. *SORL1-AS (51A)* is the antisense orientation of SORL1 intron 1 generated by alternative splicing. Upregulation of *SORL1-AS* decreases SORL1 expression by altering mRNA splicing, and by doing so, it impairs APP processing [98]. *LRP1-AS* is antisense RNA for Low-density lipoprotein receptor related protein 1 (LRP1), which disrupts LRP1-mediated Aβ clearance by directly binding to high-mobility group box 2 (Hmgb2) to block Srebp1a-dependent transcription of LRP1. Thus, *LRP1-AS* upregulation in AD brain has been mechanistically linked to the promotion of Aβ formation and decreased clearance [101, 102]. In the APPswe/PSEN1ΔE9 mouse model, *MIAT* also regulates Aβ clearance via its effects on LRP1 expression. *MIAT* downregulation promotes miR-150-5p/VEGF-mediated fibrillogenesis, decreases brain microvessel number and reduces expression of tight junction proteins. Thus, loss of *MIAT* increases Aβ40 and Aβ42 levels with a corresponding potentiation of neuronal loss in mouse model [90]. MicroRNA-106b is associated with APP generation and is downregulated in tissues from AD patients [116]. MicroRNA-200b/200c reduces Aβ secretion and insulin resistance in the brain by inhibiting ribosomal protein S6 kinase B1 (S6K1)-dependent phosphorylation of insulin receptor substrate 1 (IRS-1) to suppress the signaling of IRS-1 at serine residues [114]. MicroRNA-33 enhances Aβ clearance by promoting expression of ATP-binding cassette transporter A1 (ABCA1) and ApoE lipidation. The deletion of miR-33 has been linked to AD pathology in APPswe/PESN1ΔE9 mice [118].

Since extracellular precipitation of Aβ is promoted by its secretion, the regulation of Aβ secretion is an important factor in plaque formation. ncRNAs participate in this mechanism of regulating AD as well (Fig. 3c). Secretases are essential for Aβ secretion, and modulation of BACE-1 activity affects Aβ secretion and plaque initiation. Brain cytoplasmic 200 RNA (*BC200*) is translational regulator in postsynaptic dendritic microdomains that regulates long-term synaptic plasticity. Upregulation of *BC200* promotes BACE-1 activity and plasticity failure in AD postmortem brain tissues. Moreover, *BC200* upregulation potentiates Aβ42 expression via a direct promotion of BACE-1 expression and subsequent impairment of cell viability [84–86]. The level of neuroblastoma differentiation marker 29 (*NDM29*) is increased by inflammation. In AD patient brains, NDM29 upregulation induces APP synthesis and promotes its cleavage by BACE1 and γ-secretase. Upregulation of *BC200* or *NDM29* increase Aβ secretion and the Aβ42/40 ratio [85, 86]. *BACE1-AS* is a conserved RNA transcribed from the opposite strand of the BACE1 locus on chromosome 11 (11q23). *BACE1-AS* increases BACE1 expression by stabilizing BACE1 mRNA and prevents miR-485-5p-mediated degradation. Upregulation of *BACE1-AS* thus leads to high Aβ42 levels in AD brain tissue and mouse models [95]. *BACE1-AS* and miR-485-5p competitively regulate BACE-1, and the dysregulation of each is associated with increased BACE1 expression in AD brain sections [96]. Cyclin-dependent kinase 5 regulatory subunit 1 (CDK5R1) encodes for p35 and is an activator of CDK5. MicroRNA-15/107 negatively regulates CDK5R1 expression via direct targeting of its 3′UTR. Using AD brain sections, it was shown that CDK5R1 increases the levels of p35/CDK5, which consequently enhances CDK5 activity and modulates BACE-1 expression [111]. Further studies have linked microRNAs to reduced BACE-1 expression by direct targeting from molecules such as miR-29a/29b1/c. The downregulation of miR-29a/29b1/c leads to Aβ accumulation in peripheral blood of AD patients, postmortem brain tissues and the SAMP mouse model [117]. miR-16 can reduce BACE1 expression and attenuate Aβ-mediated neurotoxicity. miR-338-5p also regulates BACE-1 expression by targeting its mRNA, and expression of this microRNA leads to increased Aβ formation in brain tissues [122]. Downregulated miR-29a/29b1/c, miR-338-5p and miR-16 are all linked to increased Aβ-mediated neurotoxicity in vitro and in vivo [113]. Among those microRNAs, miR-338-5p also regulates NF-kB signaling and promotes neuroinflammation [122]. In vivo studies with mouse models also showed that miR-34a promotes Aβ accumulation and cognition by promoting BACE-1 expression and β-secretase activity. MiR-128 further reduces Aβ expression and inflammatory response through effects on γ-peroxisome proliferator-activated receptor (PPARγ) expression [107]. Sirt1 (also known as Sirtuin 1), a nicotinamide adenine dinucleotide-dependent deacetylase, regulates Aβ production, and miR-132/212 are correlated with Aβ production via direct targeting of Sirt 1. The downregulation of miR-132/212 fails to reduce Aβ production, and is linked to AD pathogenesis in a triple-transgenic mouse model carrying PSEN1(PS1M146V), APP (APPSwe) and Tau (TauP301L) expression constructs [108, 109].

#### ncRNAs in the regulation of tau expression and post-translational modification (PTM)

Microtubule-associated protein Tau (Tau/MAPT) is highly expressed in neurons and functions as a microtubule stabilizer. Neurofibrillary tangles comprise one of the key AD pathological signs, and are caused by hyperphosphorylation of Tau (pTau). Although the origin of pTau is still unclear, it is often observed alongside Aβ accumulation [123]. A subset of ncRNAs promotes kinase activities and thus leads to pTau (Fig. 4a). *NEAT1* has been shown to act as a microRNA decoy for miR-107, which is downregulated in AD brain tissues [125]. Upregulation of *NEAT1* corresponds to increased miR-107 activity and contributes to enhancement of Aβ expression, pTau and neuronal death [91]. MicroRNA-124 has shown neuroprotective effects, which are due to decreased BACE-1 expression and further reduction of Aβ secretion in AD mouse models [126]. *NEAT1* is also a negative regulator of miR-124 and consequently promotes BACE-1 expression. Thus, the upregulation of *NEAT1* is linked to both Aβ secretion and pTau in AD mouse models [92]. Furthermore, Tau upregulation may increase the frequency of pTau, and microRNAs have been shown to affect Tau levels to indirectly modulate pTau. MicroRNA-132/212 was shown to regulate Tau expression by directly targeting Tau mRNA in triple-transgenic AD mice. Downregulation of microRNA-132/212 is associated with GSK-3β-mediated increases in pTau [108, 110]. MicroRNA-106b targets Fyn (a Src family tyrosine kinase), which in turn, regulates Tau phosphorylation; thus, the downregulation of micRNA-106b is associated with Fyn upregulation and promotion of pTau formation [106].

#### ncRNAs in the regulation of AD-associated apoptosis

A number of ncRNA subtypes are known to regulate cellular pathways involved in AD neurodegeneration (Fig. 4b). Antisense transcript of SOX21 (*SOX21-AS*) reduces Frizzled 3/5 (FZD3/5)-mediated Wnt signaling and triggers oxidative stress generation. This increased oxidative stress subsequently promotes apoptosis in hippocampal neurons within an AD model [99]. Antisense transcript of EBF3 (*EBF3-AS*) reduces early B cell factor 3 (EBF3) mRNA expression and promotes cell death. In an AD mouse model and Aβ-treated cells, *EBF3-AS* upregulation was found to promote apoptosis [97, 103]. *NAT-RAd18* is an antisense transcript of RAd18. After Aβ40 treatment of rat cortical neurons, upregulated *NAT-RAd18* promotes DNA damage by reducing RAd18 expression, leading to cortical neuron death [100]. In a rat model with microinjection of Aβ25–35, *MEG3* is downregulated. Exogenous *MEG3* functionally protects neurons by inhibiting the PI3K/Akt signaling pathway and preventing apoptosis of hippocampal neurons. *MEG3* also decreases Aβ expression and diminishes injury from oxidative stress and inflammation [89]. Using AD postmortem tissue, it was found that *NEAT1*, *HOTAIR* and *MALAT1*, all negatively regulate the CDK5R1/p35 complex and promote cell death by controlling expression of the miR-15/107 family [93].

MicroRNAs are implicated in AD-associated apoptosis as well. Bcl2 is an anti-apoptotic protein primarily localized to mitochondria, and its expression is known to protect cell viability by opposing caspase-9-initiated apoptosis [127]. MicroRNA-34a negatively regulates Bcl2 expression through direct targeting of its 3′UTR. In AD transgenic mice, Bcl2 downregulation leads to caspase 3 activation and neurodegeneration [119, 120]. Moreover, miR-25 directly targets KLF2 and Nrf2, which are associated with hippocampal neuron proliferation. Aβ42 treatment upregulates miR-25 to promote cell death in mouse models [115]. Upregulation of Heme oxygenase 1 (HMOX1) has been linked to cognitive function and downregulation of miR183-5p in an AD mouse model. In the AD mouse, overexpressed miR-873-5p targets HMOX1 mRNA and impairs cognition. Thus, miR-873-5p has been suggested as a protective factor in the central nervous system [121]. TGF-β signaling pathway plays a key role in the AD pathogenesis, and miR-106b directly targets the 3′UTR of TGFβ-II mRNA. miR-106b-dependent TGF-β signaling reduces phosphorylation of Smad2/3 and Smad6/7 to promote neurodegeneration in APPswe/PSΔE9 AD mice [105]. Toll-like receptors (TLRs), which are innate immune receptors, have been shown to accelerate and spread central nervous system damage induced by brain-derived factors. In an AD mouse model, the RNA-sensing TLR7 is activated by extracellular let-7 and promotes neurodegeneration. Cerebrospinal fluid of AD patients contains higher amounts of let-7 compared with healthy individuals, so the microRNA let-7 has been suggested to be an important activator of TLR7-mediated damage in AD [104].

#### ncRNAs in other mechanisms of AD pathogenesis

The Alu-derived RNA, P3Alu, activates ERK1/2 signaling and the formation of the NLRP3 inflammasome in retinal pigment epithelium. Other Alu RNAs, BC200 and NDM29, are also known to be upregulated and involved in signaling during AD pathogenesis. Thus, neurodegeneration may be facilitated by Alu-induced inflammation [94]. *BDNF-AS* is a conserved noncoding antisense RNA transcript of BDNF. *BDNF-AS* represses BDNF transcription by modulating H3K27me3 at the BDNF locus and recruiting EZH2 to the BDNF promoter region. Knockdown of *BDNF-AS* restores BDNF transcription and promotes neurite outgrowth [33]. Since *NEAT1* upregulation has been linked to several signaling pathways in AD, it has been suggested as an AD biomarker and potential therapeutic target [92].

#### ncRNAs in the pathogenesis of other neurodegenerative diseases

FTLD is a common type of cortical dementia with disease onset often occurring before 65 years of age. Approximately half of all FTLD cases are associated with TDP43 inclusions, which is also a hallmark of ALS. Thus, FTLD and ALS may be categorized together as TDP43 proteinopathies [128]. The most common genetic cause of ALS and FTLD is a mutation in C9orf72, which comprises a hexanucleotide repeat expansion (HRE) GGGGCC (G4C2) in intron 1. The HRE ncRNA assembles in a G-quadruplex (G4) structure and disrupts nuclear import of proteins by sequestering GTPase activator for RAN (RANGAP1), which is essential for RAN-dependent protein/RNA nuclear import and export. Cytoplasmic accumulation of TDP43 is observed in cells with HRE and may be rescued by disrupting the HRE-RANGAP1 interaction with a cationic porphyrin, TMPyP4 [129]. HRE may also be translated into several distinct toxic dipeptides by random non-ATG-initiated translation, leading to neurodegeneration. HRE translation is regulated by the CDC73/PAF1 complex (PAF1C), a transcriptional regulator of RNA polymerase II, and this mechanism was shown to promote neurodegeneration in fly and mouse models [130]. Using tissues from postmortem ALS/FTLD patients, TDP43 was associated with upregulation of the snRNA U12 and the ncRNA *Hsrw* (stress-induced satellite III repeat RNA), both of which are linked to neurodegeneration in ALS/FTLD. Mechanistically, upregulation of U12 snRNA and *Hsrw* is caused by an interaction between TDP43 and transcription elongation factor ELL2, a shared component of the little elongation complex and super elongation complex [131]. Using iCLIP in FTLS-TDP/ALS postmortem tissues, it was also shown that *NEAT1* interacts with TDP43 and FUS/TLS [132, 133]. Since *NEAT1* is essential for paraspeckle formation, *NEAT1+* paraspeckle may be involved in ALS and FTLD etiology [134, 135]. In another example, ribonuclease angiotensin cleavage produces tiRNAs from tRNAs, which assemble as G4 structures and inhibit translation by displacing eIF4F from capped mRNA. Translational repressor Y-box 1 (YB-1) interacts with and is stabilized by tiRNAs. This interaction leads to stress granule formation and promotes motor neuron degeneration [136]. In ALS, the antisense transcript of CCND1 (ncRNACCND1) represses CCND1 transcription by recruiting FUS/TLS to the CCND1 promoter domain and inhibiting CREB-binding protein and p300 histone acetyltransferase activities. FUS/TLS mislocalization is a phenotype of the FUS-related ALS subtype, and CCND1 is important in DNA damage response. As such, ncRNACCND1-mediated CCND1 downregulation is linked to DNA damage and apoptosis in cellular models of FUS-related ALS [137].

Furthermore, dysregulated ncRNAs (*Lhx1as*, *lncMN-1*, and *lncMN2*) have been identified in FUS-P517L ALS mouse models, but the consequences are unknown [138]. CircRNAs are also known to be dysregulated in FUS-mutated cells and motor neurons derived from ALS-iPSCs, but the functional roles of those circRNAs are still unclear as well [139]. In samples from ALS patients, the profiles of ncRNA expression shed light on how ALS pathogenesis may vary based on the genetic basis of disease. NATs provide a good example of how such insights may be gained. In FUS-mutated patients, *PAXBP-AS* is the antisense of PAX3/PAX7 binding protein (PAXBP), which is a transcriptional factor that regulates chromosome binding. *PAXBP-AS* regulation of PAX3/7 pathways has been implicated in developing FUS-ALS. In ALS-TDP43 patients, *SNAP25-AS* is downregulated compared with healthy subjects. Because SNAP25 regulates synaptic vesicle processing and axonal repair, these cellular processes are likely to be negatively affected by TDP43 mutation in ALS motor neurons. In ALS-SOD1, CKMT2, a mitochondrial creatine kinase (MtCK), and its antisense transcript (*CKMT2-AS*) have been linked to mitochondrial dysfunction and subsequent motor neuron degeneration. In sporadic ALS, IER3-AS is involved in regulating cell survival via promotion of NFκB expression; NFκB regulation of apoptosis is thought to be important in sporadic ALS. *ZBTB11-AS* is potentially involved in DNA binding and regulation of transcription based on the known function of its target, Zinc Finger and BTB Domain containing 11 (ZBTB11) [140]. Although the functional impacts of the aforementioned antisense transcripts are still unknown, the regulatory pathways that the corresponding sense RNAs contribute to offer starting points for investigations into the pathogenic mechanisms of ALS [140].

MicroRNAs have been implicated in ALS pathogenesis as well. Cytoplasmic neurofilament aggregation is a major characteristic of ALS, and misregulated neurofilament proteins may serve as a potential diagnostic biomarker for ALS [141]. Upregulation of NEFM (neurofilament triplet M protein) and NEFH (neurofilament triplet H protein) has been detected in ALS spinal cord tissues. NEFM is reduced by miR-92a-3p and miR-125b-5p, and the *NEFH* 3′UTR is targeted by miR-124-3p, miR-92a-3p, miR-20b-5p and miR-223b-3p. The downregulation of miRNAs alters the stoichiometry of neurofilament expression and leads to the formation of pathogenic aggregates in ALS [142]. Tumor suppressor gene NDRG2 and miR-375-3p are dysregulated in a sporadic ALS mouse model carrying the Vps54 mutation. Upregulated NDRG2 increases reactive oxygen species formation and promotes p53 activity. Interestingly, insufficient targeting of p53 by miR-375-3p leads to upregulation of NDRG2 and reactive oxygen species production [143]. In FUS^P525L^ motor neurons, miR-375 serves to protect motor neurons from apoptosis through targeting of p53 and ELAVL4 [144]. Importantly, several studies have shown that microRNAs can alter lifespan in ALS/FTLD laboratory models. For instance, AAV-mediated expression of the miR17–92 cluster prolongs lifespan of SOD1^G93A^ mice. This cluster targets an E3 ubiguitin ligase to regulate monoubiquitination of PTEN, which determines the subcellular location of the protein. By this mechanism, miR17–92 is able to regulate motor neuron vulnerability in ALS [145, 146]. In another example, treatments to suppress miR-155, which is upregulated in ALS model rodents and patient spinal cord, improve survival in SOD1^G93A^ rodent models mainly by blocking miR-155 function in microglia, astrocytes and neurons [22, 147]. Another study in ALS-SOD1^G93A^ rodent models showed that miR-155 regulates microglia expression of survival genes, including P2ry12, Tmem119, Olfml3, Egr1, Atf3, Jun, Fos, Mafb and Tgfbr1 [22]. Antisense oligonucleotides of miR-155 successfully inhibit miRNA function in spinal cord and brain, and improvements in survival time suggest that miR-155 may be a viable therapeutic target for ALS. Furthermore, microRNAs may be transported in extracellular vesicles and potentially spread pathogenic factors to neighboring cells. In ALS, upregulated miR-218 is transported from motor neurons to neighboring astrocytes, where it is sufficient to downregulate glutamate transporter, excitatory amino acid transport 2 (EAAT2). Blocking miR-218 with antisense oligonucleotides rescues EAAT2 expression and mitigates astrogliosis in mouse brain [148]. Neuromuscular synapses between motor neurons and muscle tissue are essential for motor neuron and muscle function. Exogenous expression of miR-206 slows ALS progression by regulating histone deacetylase 4 expression and fibroblast growth factor signaling. In response to motor neuron injury, miR-206 can promote a compensatory response in neuromuscular synapses and restore the function of neuromuscular junctions [149]. Spinal muscular atrophy (SMA) is a group of neuromuscular diseases caused by genetic mutations that compromise survival of motor neurons. U1 snRNA (U1) is essential for regulating pre-mRNA splicing, and its variant snRNAs (vU1s) are inversely correlated with U1 expression. The ratio of vU1/U1 is dysregulated in SMA-iPSC-derived motor neurons compared with healthy controls. Upregulated vU1 snRNA is also observed in SMA-subjects, and thus, vU1 may contribute to SMA pathogenesis [150]. Spinocerebellar ataxia type 2 (SCA2) is caused by a CAG expansion in ataxin-2 (ATXN-2). Interestingly, *ATXN2-AS* transcripts with CAG repeats form RNA foci that induce caspase3/7-dependent apoptosis in cerebellar Purkinje cells [151].

Collectively, this growing body of studies shows that ncRNAs contribute to neurodegeneration in many types of dementia and lower motor neuron disease. Thus, ncRNA-based treatments may have potential to improve lifespan and disease symptoms. The ncRNAs known to participate in the aforementioned diseases are summarized in Table 4. Although there are still no effective drugs for any of these diseases, promising results from animal models can provide potential targets for future therapeutic development.

**Table 4:** ncRNAs and related processes in other neurodegenerative diseases

Disease	ncRNA class	Name	up/down regulation	Mutation	Description	Model	Ref
ALS	**NATs**	*IER3-AS, ZBTB11-AS, PAXBP-AS, SNAP25-AS, CKMT2-AS,*	down, down, up,down, down	SALS, TDP43, SOD1, FUS	*IER3-AS* and *ZBTB11-AS *downregulated in sporadic ALS; *PAXBP-AS* upregulaed in ALS-FUS; *SNAP25-AS *downregulated in ALS-FUS;* CKMT2-AS* downregulated in ALS-SOD1. The mechnisms are still unkown.	ALS spinal cord extract, peripheral blood mononuclear cells	[140]
	**LncRNAs**	ncRNA_CCND1_	up	FUS/TLS	In response to DNA damage, ncRNA_CCND1_ interactes with FUS and represses CCND1 transcription by enhancing inhibition of CBP and p300 histone acetyltransferase activities.		[137]
		tiRNAs (tRNA-derived RNA fragments)	up	ANG-P112L	tiRNAs inhibit translation via its G-guadruplex structure. tiRNAs displace eIF4 from mRNA and stablizes YB-1. tiRNAs promotes the untranslated mRNA for stress granule formation.	cell line	[136]
		Lhx1as, LncMN-1, LncMN2		Fus P517L	detected in mouse model without know mechanisms.	mouse	[138]
	**MicroRNAs**	miR-17~92	down	SOD1^G93A;^ SOD1^L144F^	miR-17~92 cluster target E3 ubiguitin ligase to regulate PTEN subcellular location via monoubiquitination. miR-17~92/nuclear PTEN regulats motor neuron vulnerability in SOD1ALS.	ALS patienst’s iPSC, mouse model	[145, 146]
		miR-155	up	SOD1^G93A^	miR-155 distributes in rodent and patients’ spinal cord. Anti-miR-155 treatement improve survival rate by mainly blocking miR-155 funcion in microglia, astrocyte and neuron.	ALS spinal cord, rodent model	[147]
		miR-155	up	SOD1^G93A^	miR-155 regulates survival gene expression in microglia incuding P2ry12, Tmem119, Olfml3, Egr1, Atf3, Jun, Fos, and Mafb and Tgfbr1 .	ALS spinal cord, rodent model	[22]
		miR-206	down		miR-206 contols HDAC4 expression in neuromuscular gene expression and restore the NMJ function.	mouse model	[149]
		miR-218	up	SOD1^G93A^	miR-218 can be transported from motor neurons to neighbouring astrocytes and sufficiently downregulates glutamate transporter in astrocytes (excitatory amino acid transport 2 (EAAT2)). Blocking miR-218 with antisense oligonucleotides recover EAAT2 expression and mitigates astrogliosis in mouse brain.	mouse model	[148]
		miR-375-3p	down	Vps54	tumor suppressor gene NDRG2 and miR-375-3p are dysregulated in sporadic ALS. Upregulated NDRG2 increas ROS formation and further activates p53. Insufficient targeting p53 by miR-365-3p leads to NDRG2 and ROS upregulation.	ALS-iPSC	[143]
		miR-375	down	FUS^P525L^	miR-375 targets p53 and ELAVL4, which are upregulated due to loss of FUS function.	ALS-iPSC	[144]
		miR-92a-3p, miR-125b-5p,	down		miR-92a-3p and miR-125b-5p target NEFM 3’UTR .	ALS spinal cord	[142]
		miR-124-3p, miR-92a-3p, miR-20b-5p miR-223b-3p,	down		miR-124-3p, miR-92a-3p, miR-20b-5p and miR-223b-3p target NEFH 3’UTR.	ALS spinal cord	[142]
ALS; FTLD	**LncRNA**	C9ORF72 (repeat expansion)	up	hexanucleotide repeat expansion in C9orf72 intron 1	HRE repeats expansion disrupts RAN dependent protein/RNA nucleaocytoplasmic transport by sequestering RNAGAP1 and leads to neurodegeneration. HRE also get translated into toxic dipeptide by interacting with PAC1 depedent translation factor and leads to neurodegeneration.	ALS patients’ brain, spinal cord; iPSC, mouse, fly	[129, 130]
		*MALAT1, MEG3*		TDP43	In iCLIP data, *MALAT1* inteactes with TDP43, and FUS interactes with *MEG3.*	ALS patinets’ tissue extract	[132, 133]
		*NEAT1*	up	TDP43, FUS	*NEAT1_2* interacte with TDP43 and FUS by iCLIP data. TDP43 and FUS are recruited to paraspeckle due to interaction with *NEAT1_2*.	ALS patients’ tissue extract	[132–135]
	**snRNA**	*U12 snRNA, Hsrw*	up	TDP43	associated with neurodegeneration caused by TDP43 promoted transcrption elongation via interaction with ELL2 in elongation complexes.	Fly	[131]
SMA	**snRNA**	variant of U1 snRNA (vU1)	up		variant of U1 snRNA (vU1) is upregulated and affects U1 snRNA expression. The ratio of vU1/U1 increased in SMA-iPSC derived MN compared with control.	SMA-iPSC	[150]
SCA2	**NATs**	*ATXN2-AS*			*ATXN1-AS* transcripts with CAG repeats forms RNA foci and detected in SCA2 cerebellar Purkinje cell. ATXN1-AS and CAG repeats trigger caspase 3/7 dependent apoptosis.	SCA2 tissues	[151]

## Conclusions and future perspectives

Accumulating evidence demonstrates that ncRNAs play important roles in the central nervous system and regulate neurodegeneration. In this work, we have summarized recent studies elucidating the varied roles of ncRNAs in neurodegenerative diseases, including HD, PD, AD, ALS, FTLD, SCA2 and SMA. Development of ncRNA-based treatments for neurodegenerative diseases may represent a novel and potentially effective therapeutic strategy, which could also further our understanding ncRNA biology [152]. Recent studies on ncRNAs in neurodegenerative diseases have mostly focused on miRNAs as decoys [12] and/or transcriptional regulators [25, 30, 153]. Systems biology and bioinformatics approaches will be required to unravel the functional roles of a wider class of ncRNAs and complex RNA networks in each disease state [154]. Moreover, valuable insights into the spatial context of RNA expression may be gained by novel technologies, such as single-cell sequencing [155] and spatial transcriptome sequencing [156]. Finally, novel drug delivery technologies can be developed to effectively target ncRNAs to specific regions [157]. For example, oligonucleotide modifications can be used to improve targeting and pharmacokinetics of RNA-based drugs [158]. Although secondary structure of ncRNAs is presumably an obstacle to their development as drugs, chemically modified analogues might be used to overcome the limitation [159]. Hopefully, ncRNA-based treatments will someday be realized as new medicines to prevent onset and extend survival in patients with devastating neurodegenerative diseases.

## Abbreviations

6-OHDA: 6-hydroxy-dopamine
ABCA1: ATP-binding cassette transporter A1
Abhd11-os: MGI Symbol
APP: Amyloid precursor protein
ATP13A2: ATPase type 13A
BACE1: β-site amyloid precursor protein-cleaving enzyme 1
BC200: Brain cytoplasmic 200 RNA
BDNF: Brain-derived neurotropic factor
BDNF-AS: Brain-derived neurotrophic factor antisense
CACNG5: Calcium voltage-gated channel auxiliary subunit gamma 5
CBP: CREB-binding protein
CCCP: Mitochondrial oxidative phosphorylation uncoupler
CCNA2: Cyclin-A2
CDK5R1: Cyclin-dependent kinase 5 regulatory subunit 1
CHEK1: Checkpoint kinase 1
CoREST: REST corepressor 1
DGCR5: DiGeorge syndrome critical region gene 5
EAAT2: excitatory amino acid transport 2
EBF3: B cell factor 3
EBF3-AS: Antisense transcript of EBF3
FMRP: fragile X syndrome protein
Fyn: a Src family tyrosine kinase
FZD3/5: Frizzled 3/5
GDNF: Glial cell-derived neurotrophic factor
GLAST: L-glutamate/L-aspartate transporter
GLT1: Glutamate transporter subtype 1
GPR51: G-protein-coupled-receptor 51
HAR1: Human accelerated region 1
HDAC4: Histone deacetylase 4
HIP1: Huntingtin Interaction Protein 1
Hmgb2: High-mobility group box 2
HMOX1: Heme oxygenase 1
HOTAIR: Hox transcript antisense intergenic RNA
Hsrw: Stress-induced satellite III repeat RNA
*HTT*: Hungtingtin
HTT-AS: Huntingtin antisense
IRS-1: Insulin receptor substrate 1
IRS-1pSer: The insulin receptor substrate 1 at serine residues
KLF2: Kruppel-like factor 2
LPS: Lipopolysaccharide
LRP1: Low-density lipoprotein receptor related protein 1
LRRK2: Leucine-rich repeat kinase 2
MDR1: Multidrug resistance protein 1
MEG3: Maternally expressed 3
mHTT: Mutant HTT
MPP +: methyl-4-phenylpyridinium
MPTP: 1-methyl-4-phenyl-1236-tetrahydropyridine
MSN: Striatal medium spiny neurons
MtCK: Mitochondrial creatine kinase
NAT-RAd18: Antisense transcript of RAd18
NATs: Natural antisense transcripts
ncRNACCND1: Antisense transcript of CCND1
NDM29: Neuroblastoma differentiation marker 29
NEAT1: Nuclear Enriched Abundant Transcript 1
NEFH: Neurofilament triplet H protein
NEFM: Neurofilament Triplet M Protein
NLRP3: Nod-like receptor protein 3
NORAD: Noncoding RNA activated by DNA damage
Nrf2: Nuclear factor erythroid 2-related factor 2
PAF1C: CDC73/PAF1 complex
PAK1: p21-activated kinase 1
PARK2: Parkin
PARK7: Protein deglycase DJ-1
PAXBP: PAX3/PAX7 binding protein
PCNA: Proliferating cell nuclear antigen
PCR2: polycomb repressive complex 2
PGC-1α: Peroxisome proliferator-activated receptor-γ coactivator
PINK1: PTEN-induced kinase 1
PPARγ: γ-peroxisome proliferator-activated receptor
PPX: Pramipexole
PRC2: Polycomb repressive complex 2
RANBP10: RAN binding protein 10
REST: Repressor Element 1-Silencing Transcription factor
RGS2: Regulator of G-protein signaling 2
RhoA: Ras homolog family member A
S6K1: Ribosomal protein S6 kinase B1
SAMP mouse model: A naturally occuring mouse line that displays a phenotype of accelerated aging
sCAGs: small CAG-repeated RNAs
Sirt1: Sirtuin 1, a nicotinamide adenine dinucleotide-dependent deacetylase
SNCA: α-synuclein
SNHG1: Small nucleolar RNA host gene 1
SORL1-AS (51A): Antisense of sortilin related receptor 1
TDP43: TAR DNA BINDING PROTEIN
TH: Tyrosine hydroxylase
TLR: Toll-like receptor
TRPM2: Transient receptor potential cation channel subfamily M member 2
TRPM7: Transient receptor potential cation channel subfamily M member 7
TUG1: Taurine Upregulated Gene 1
TUNA: Tcl1 Upstream Neuron-Associated lincRNA
UCHL1: Ubiquitin carboxy terminal hydrolase L1
ZBTB11: Zinc Finger and BTB Domain containing 11
